# Anatomy of Nutrient Foramina of Adult Humerii in the Pakistani Population: A Cross-Sectional Study

**DOI:** 10.7759/cureus.19052

**Published:** 2021-10-26

**Authors:** Muhammad Haris, Sobia Haris, Farah Deeba, Muhammad Jehangir Khan

**Affiliations:** 1 Department of Anatomy, Nowshera Medical College, Nowshera, PAK; 2 Medical Education and Simulation, Nowshera Medical College, Nowshera, PAK; 3 Department of Pediatric Surgery, Nowshera Medical College, Nowshera, PAK

**Keywords:** nutrient artery, nutrient foramen, humerus, anatomy, adult

## Abstract

Objective

In this study, we aimed to analyze the anatomy of adult humerii nutrient foramina in the Pakistani population, including the number, size, and relative position of the nutrient foramen in relation to the outer surface and zones, as well as length from the center of the humerus.

Materials and methods

Dry humerii of unknown age and gender were included and analyzed through non-probability convenience sampling. Those that were broken or had any pathology were excluded. The length of the humerus (cm), the number, size, and position of the nutrient foramen in relation to humerus surfaces and zones, as well as the distance from the humerus midpoint were studied. When many foramina were identified, the largest was designated as prominent foramen, and its dimensions (mm) were calculated. The data were collected and analyzed, i.e., mean, range, percentage, and standard deviation.

Results

A total of 50 adult dry humerii of unknown age and gender were studied. The humerii had a mean length of about 27.96 ±2.18 cm. The nutrient foramen had a mean size of about 0.828 ±0.26 mm. The mean distance from the humerus center to the major nutrient foramen was nearly 2.31 ±1.25 cm. The nutrient foramen was discovered in the bone in the middle one-third of humerii (84%) and 12% in the lower one-third, while it was only detected in 4% in the top one-third. The nutrient foramen was located in the anteromedial surface 80% of the time, the posterior surface 12% of the time, and the anterolateral surface 8% of the time.

Conclusion

Based on our findings, the nutrient foramina of adult humerii in the Pakistani population studied were discovered in the anteromedial and posterior surfaces on the anterolateral. Additionally, the nutrient foramen was identified in the middle and lower thirds of the humerii. The majority of the humerii had only one nutrient foramen, while a few humerii had several nutrient foramina. We believe physicians will find our results useful in treating humeral injuries and illnesses.

## Introduction

Foramina for blood vessel entrance vary in size and shape in all bones [1]. Very large nutritional foramina are found in the shaft of the bone with larger lengths, feeding into a nutrient canal that continues into the medullary cavity [1]. The nutrition foramen is oriented outwards from the bone's developing terminal [1]. The developing points of bones in the upper limb are the upper tips of the humerus and the lower tips of the radius and ulna [1].

As the incidence of commercial and transportation accidents, athletic injuries, and pathological cracks in osteoporotic individuals rises, long bone fractures are becoming more common [2]. A closed or open reduction may cause a broken bone to fail to mend [2]. The fracture may become delayed or result in nonunion if the blood supply is inadequate, indicating that the medullary artery system is essential in the reformation of the vessels of the necrotizing cortex and the union of callus at ruptures spot [2]. Surgeons can avoid harming the nutrient artery and limiting the development of a late union or nonunion of the crack by recognizing the position of the nutritional foramen and the accompanying anatomy [3].

The nutrient foramen is the entry point for the nutrient artery to give nourishment in various areas of the bone, which plays a very important role in the healing process after fracture, trauma, and bone grafting [4]. The nutritional vessels keep the medulla alive and nourish the inner part of the cortex. When just the nutrient channels are intact, the repair is active [5]. The metaphyseal vessels remain viable throughout the medulla and the inner half of the cortex, although healing is not as vigorous as in controls except around the metaphyseal ends, and is significantly delayed in the shaft's middle area [5,6]. The blood supply to bone is well characterized, which aids the reconstructive surgeon in the design of vascularized bone to be appropriate for transfer into defects needing bone substitution [7].

The purpose of this research was to study the anatomy of adult humerii nutrient foramina in the Pakistani population, including the number, size, and relative position of the nutrient foramen in relation to the outer surface and zones, as well as length from the center of the humerus.

## Materials and methods

Study design and setting

This was a descriptive cross-sectional study conducted at the Department of Anatomy, Nowshera Medical College (NMC), Nowshera, Pakistan.

Study duration, sample size, and sampling technique

After obtaining ethical approval from the Institutional Ethical Review Board (IERB) at NMC vide its letter No: 20/IERB/NMC/Sec dated 14/12/2020, this study was conducted in June 2021. A total of 50 adult dry humerii were included and studied. Non-probability convenience sampling was employed in the analysis.

Sample selection

All adult dry humerii of unknown age and gender available in the anatomy museum were included in the study, whereas those that were broken or had any pathology were excluded.

Data collection

All adult dry humerii, not necessarily coupled, as well as those of undetermined age and sex, were investigated. The measurement of the humerus, count and size of the nutrient foramen, its position relative to zones and surfaces, and its distance from the humeral midpoint were all measured. Moreover, zone I (upper third), zone II (middle third), and zone III (lower third) were used to split the humerus bone into three equal zones. The midpoint of the humerus was located and marked on the humerus using an osteometric board after dividing its length by two. The nutrient foramen was identified in connection with three surfaces: anteromedial, anterolateral, and posterior, as well as three zones: zone I, zone II, and zone III. A vernier caliper was used to calculate the length between the nutritional foramen and the center of the humerus. Hypodermic needles with known diameters ranging from 18 to 26 gauge were utilized to calculate the size of the nutritive foramen (18 gauge = 1.2 mm, 20 gauge = 0.9 mm, 24 gauge = 0.55 mm, and 26 gauge = 0.45 mm). After identifying the number of foramina, the biggest nutrient foramen were chosen as the main foramen, and the magnitude was recorded.

Data analysis

All of the data were collected, and the percentage, mean, and standard deviation were computed as part of the descriptive statistical analysis.

## Results

A total of 50 adult dry humerii of unknown age and gender were investigated, with 56% (28) of the bones on the left side and 44% (22) of the bones on the right side. The average humerii length was about 27.96 ±2.18 cm (range: 18-33 cm). The average measurement of the length was 28 cm. Although a few humerii had more than one nutrient foramina, the majority of humerii had only one (Table 1).

**Table 1:** Nutrient foramen count in humerii (n=50)

Number of foramina	Number of humerii	Percentage
1	33	66%
2	14	28%
3	3	6%

Foramina diameters ranged between 0.45-1.2 mm, with an average of about 0.828 ±0.26 mm. The measurement of length from the middle of the humerus to the prominent nutrient foramen varied from 0.5-8 cm, with an average value of about 2.31 ±1.25 cm. Of note, 88.76% of the nutrient foramina were discovered above the humerii midpoints, whereas 11.24% were found below the humerii midpoints. The majority of the nutrient foramina were found on the humerii anteromedial surfaces (Table 2). In the mainstream of the humerii, the nutrient foramina were identified in zone II (one-third from the middle) of the bone as shown in Table 3.

**Table 2:** Location of the number of nutrient foramina in relation to the surface of the humerus (n=50)

Location of foramen	Number of humerii	Percentage
Anteromedial surface	40	80%
Anterolateral surface	4	8%
Posterior surface	6	12%

**Table 3:** Number of nutrient foramen with regard to zones of the humerus (n=50)

Zone	Number of humerii	Percentage
Zone I	2	4%
Zone II	42	84%
Zone III	6	12%

## Discussion

The blood flow is the most important component in fracture curing [4-6]. Any injury occurring to the nutrient artery throughout surgical fixation or successive handlings constitutes a major risk for nonunion or poor wound healing [8-11]. The nutrient artery, the metaphyseal artery, as well as periosteal arteries from the axillary and brachial arteries, all nourish the humerus [12]. Blood is supplied to the outer cortex and metaphysis of the bone through the periosteal and metaphyseal arteries, while the nutrient artery provides blood to the shaft medulla and the internal half of the cortex [12]. The study of blood flow of the shaft will help researchers better comprehend fracture healing, late unions and malunions of bone after fractures, and bone transplantation [12].

Mansur et al. analyzed humeral vascularity in their study and found that the primary nutrient artery of the humerus should be shielded from damage throughout humeral shaft operations [7]. According to Xue et al., the nutrient artery enters the humerus through the controlled anteromedial surface, while also entering the middle one-third of the shaft, and surgeries performed on the middle one-third of the shaft of the humerus must be performed carefully to avoid damaging the nutrient foramen, so as to avoid late unions or malunion of the bones [3]. Our findings were consistent with those of Xue et al. [3]. Many humerus bone examinations have indicated that a huge number of the humerii have a nutrient artery and others have extra-auxiliary arteries. The findings of our research corresponded well with some studies but differed from some others, and this might be due to differences among the study population [3,7,13,14]. The humerii had one or two major nutritional foramina, which were directed towards the elbow and sandwiched by the coracobrachialis and brachial muscles with an average diameter of 1.11 ±0.32 mm. The mean index was 43.76 ±4.94%, and the landmark index was 42.26 ±5.35%. No noticeable changes were found in diameter, length, or nutrition foramina index between the two sides. Also, no significant relationships were found among transverse and longitudinal positions or between full length and diameter. The foramina index and the landmark index showed a substantial positive connection (r=0.994, p<0.0001) [3].

According to another study, 60.87% had a single nutrient foramen, 28.85% had a twofold foramen, 6.32% had a triple foramen, and 1.98% of the humerii had four nutrient foramina, while 1.98% had none [7]. The bulk of the nutritional foramina (88.86%) were found on the anteromedial surface, 6.52% on the anterolateral surface, and 4.62% on the posterior side of the humerii shaft. The majority (94.84%) of the foramina was found in zone II, followed by zone III (4.62%), and finally zone I (0.54%). All foramina were oriented at the humerii distal end [7]. The position and quantity of nutritional foramina in the diaphysis of 885 long bones from limbs of adults including upper and lower limbs were investigated in this study, the details of which are as follows: 174 humerii, 157 radii, 146 ulnae, 152 femora, 142 tibiae, and 114 fibulae. The nutritional foramina were present on the anterior aspect of the upper limb long bones and the posterior portion of the lower limb long bones. A single feeding foramen was found in the majority of the bones tested, which might imply a single source of blood supply. The humerus had a foramina index of 55.2%; the same index for radius was 35.7%, and for ulna, it was 37.9%, while the femur had a foramina value of 43.7%; it was 32.7% for tibia, and 46.1% for fibula.

The present research gathered information on the populace of the southern area of Brazil, offering ethnic data for evaluation and perhaps aiding surgical techniques and radiological image interpretation [13]. The nutrient was single in 90.62% of the bones, double in 7.8%, and missing in 1.56%. In 65.62% of humerii, the most foramen was observed on the anteromedial surface, followed by the medial border in 21.87% of humerii. The majority of the foramen (81.25%) was found in the middle part of the humerus' diaphysis. In all humerii, the direction of the nutritional foramen was distal [14]. According to Kumari et al. and Hansen, there is no relationship between humeral length and the number of nutrient foramina [14,15]. And hence, we did not attempt to link the two. This work adds to the current evidence that population diversity can result in a difference in several nutrient foramina.

## Conclusions

Our findings have revealed that the nutrient foramina of adult humerii in the Pakistani population were discovered in the anteromedial and posterior surfaces on the anterolateral. Also, the nutrient foramen was identified in the middle and lower thirds of the humerii. The majority of the humerii had only one nutrient foramen, whereas a few humerii had several nutrient foramina. Our research provides significant insight into the nutrient foramina, which will be useful to doctors treating humeral injuries and illnesses. Knowing the number and position of nutrient foramina in the humerus will help prevent nutrient artery damage during orthopedic surgeries, and prove to be valuable in medico-legal practice.
